# Complementary Feeding and Iron Status: “*The Unbearable Lightness of Being*” Infants

**DOI:** 10.3390/nu13124201

**Published:** 2021-11-23

**Authors:** Vito Leonardo Miniello, Maria Carmen Verga, Andrea Miniello, Cristina Di Mauro, Lucia Diaferio, Ruggiero Francavilla

**Affiliations:** 1 Nutrition Unit, Department of Pediatrics, “Giovanni XXIII” Children Hospital, “Aldo Moro” University of Bari, 70126 Bari, Italy; 2 Primary Care Pediatrics, ASL Salerno, 84019 Vietri sul Mare, Italy; vergasa@virgilio.it; 3 Department of Allergology and Immunology, “Aldo Moro” University of Bari, 70124 Bari, Italy; miniello_andrea@yahoo.it; 4 Regional Centre of Pharmacovigilance Campania, Department of Experimental Medicine, University “Luigi Vanvitelli”, 80138 Naples, Italy; cristinadimauro@live.it; 5 Giovanni XXIII” Children Hospital, 70126 Bari, Italy; luciadiaferio83@gmail.com; 6 Gastroenterology Unit, Department of Pediatrics, “Giovanni XXIII” Children Hospital, “Aldo Moro” University of Bari, 70126 Bari, Italy; ruggiero.francavilla@uniba.it

**Keywords:** complementary feeding, iron, iron deficiency, iron deficiency anemia, infants

## Abstract

The complementary feeding (CF) period that takes place between 6 and 24 months of age is of key importance for nutritional and developmental reasons during the transition from exclusively feeding on milk to family meals. In 2021, a multidisciplinary panel of experts from four Italian scientific pediatric societies elaborated a consensus document on CF, focusing in particular on healthy term infants. The aim was to provide healthcare providers with useful guidelines for clinical practice. Complementary feeding is also the time window when iron deficiency (ID) and iron deficiency anemia (IDA) are most prevalent. Thus, it is appropriate to address the problem of iron deficiency through nutritional interventions. Adequate iron intake during the first two years is critical since rapid growth in that period increases iron requirements per kilogram more than at any other developmental stage. Complementary foods should be introduced at around six months of age, taking into account infant iron status.

## 1. Introduction

As a cross-cutting area in the health and development sectors, nutrition is an integral part of the World Health Organization (WHO) 13th General Program of Work (GPW13) 2019–2023 [1]. Nutrition science provides strong evidence to support the concept of the “life-course approach”, which focuses on how multiple determinants interact to influence health and well-being throughout life [2]. From conception to adulthood, nutrition is of profoundly significant importance in influencing individual or population health, either positively or negatively, across the lifespan and even for generations to come.

There is growing scientific evidence that nutrition in early childhood is associated with the risk of developing non-communicable diseases (NCDs). These include obesity, dyslipidemia, diabetes, hypertension, cardiovascular diseases, chronic lung diseases, cancer, and musculoskeletal, mental, and neurological disorders [3]. The term NCDs has been extended to include other chronic health conditions, such as renal, hepatic, gastroenterological, endocrine, hematological, dermatological, and genetic diseases [4]. It is now well accepted that the risk of developing various NCDs begins in infancy or childhood and probably even during fetal development.

Nutrition is one of the easily modifiable environmental factors that can affect growth, development, infant metabolism, and the immune system. It is an example of phenotypic plasticity, allowing a genotype to give different physiological states.

The so-called “first thousand days of life” (roughly spanning between conception and the second birthday) is a critical and crucial period in which the foundations are laid for healthy growth and neurological development across the lifespan; it is of central importance because it allows us to positively affect the child physical and cognitive development [5].

Iron deficiency (ID) and iron deficiency anemia (IDA) are still global problems today. The prevalence of non-anemic iron deficiency is three times higher than the prevalence of IDA. It should be noted that a persistent ID with or without anemia is associated with neurocognitive consequences, which can be challenging to reverse entirely even in the case of iron supplementation [6,7].

The complementary feeding phase (CF), when foods are introduced to complement milk feeding, is generally between 6 and 23 months of age. This period is characterized by rapid development and growth, exposing infants to an increased risk of nutrient excesses or deficiencies, especially ID and IDA, which are most common at this age [8]. Consequently, complementary foods (semisolids, solid foods and liquids other than breast milk, infant formula, follow-on formula, and young-child formula) and correct feeding practices can prevent malnutrition.

In 2021 a multidisciplinary expert panel of four Italian scientific pediatric societies produced a document on CF. The aim was to provide useful clinical advice for pediatricians working in Pediatric Divisions, Primary Care Services, residents or Ph.D. students, pediatric nurses, and specialists. The nutrition committees of the Italian Society for Preventive and Social Pediatrics (SIPPS), the Italian Society for Developmental Origins of Health and Disease (SIDOHaD), the Italian Federation of Pediatricians (FIMP), and the Italian Society of Pediatric Nutrition (SINUPE) provided an update of the available clinical literature.

## 2. Iron Status before the Introduction of Complementary Feeding

Fetal and neonatal (perinatal) periods and infancy are considered susceptible periods in the course of life when exposure determines or reduces the risk of disease [9]. This susceptibility is determined by the increased demands caused by rapid growth, which are often not met by nutrition. Therefore, early nutrition continues to be the center of scientific attention as a factor determining growth and development as well as health and neurological development later in life.

Scientific research advances our understanding of the underlying mechanisms contributing to iron deficiency and helps us identify and introduce new targeted strategies. Metals such as iron, zinc, copper, and manganese are recognized as essential trace elements. In the human body, most trace elements are bound to proteins. Iron and iron-containing compounds (iron cluster proteins and hemoproteins) play an essential role in biological processes that are essential to the survival and function of living organisms. Metalloproteins can bind iron directly or use iron-containing complexes such as heme or iron-sulfur (Fe-S) clusters. These proteins have diverse and essential functions within the cell, including carrying oxygen (hemoglobin), oxygen storage (myoglobin), cellular metabolism (amino acid oxidases, fatty acid desaturases), energy production (cytochrome-c), detoxification (catalase), and host defense (myeloperoxidase contained in the intracellular granules of neutrophils, NADPH oxidase, responsible for the oxygen burst) [10]. Iron is crucial for immune metabolism [11,12], appropriate innate and adaptive immune responses, T cell activation and differentiation (T cell development, Th1/Th2 polarization), behavior and neurodevelopment (including myelination, oligodendrogenesis, synaptogenesis, and neurotransmission) [13], ATP production, #protein, and DNA synthesis and ferroptosis (an iron-dependent, non-apoptotic modality of cell death that is characterized by the accumulation of lipid-reactive oxygen species) [14].

Due to its electrochemical properties, iron can act as a redox-active cofactor in many different biological processes. Because of the ability of ferrous iron (Fe^2+^) to reduce intermediate oxygen species into harmful free radicals, iron can be considered a “double-edged sword” [15].

The interpretation of iron status indices measured in healthy infants can be particularly difficult. The first biochemical evidence of depleted tissue iron stores is low serum ferritin (<12 μ/L in infants and young children). There may be confounding interferences because serum ferritin is elevated during inflammation or infection (acute phase effect); in contrast, serum iron concentration is reduced in these scenarios. After a more prolonged period of iron deficiency, ferritin depletion is followed by a shortage of iron-containing proteins/enzymes: a reduction in circulating transferrin iron saturation increased circulating transferrin protein concentration, circulating transferrin receptor concentrations, and free erythrocyte protoporphyrin in the red cells. Finally, the last step in the depletion sequence is overt anemia, with decreased hemoglobin concentration.

Iron deficiency and the resulting anemia can have dangerous functional consequences, especially for the developmental indices of infants and young children. The American Academy of Pediatrics (AAP) concluded that IDA negatively affects an infant’s behavioral development and cognitive performance [16]. Recently, Stoffel et al. found that ID/IDA at the time of infant vaccination can impair the response to diphtheria and pertussis vaccines. Optimizing the iron status can improve response to the measles vaccine [17].

Prevention of ID is as important as treating an existing deficiency, especially if it is severe enough to cause IDA.

Approximately half the iron needed for infant growth and development should generally be absorbed before birth by the mother during the third trimester of pregnancy. Therefore, most healthy term infants have adequate iron stores at birth to meet their six-month needs. Iron stores become gradually depleted at about this time, and breast milk alone can no longer meet the infant’s iron requirements. The WHO and the Pan American Health Organization (PAHO) recommend exclusively breastfeeding for the first six months of life, in combination with appropriate CF beyond this age [18]. Although exclusively breastfeeding is recommended for the first 6 months, we have to keep in mind that the iron content of breast milk is low. In contrast, infant formulas contain higher iron contents than breast milk, even though iron absorption is approximately 20–50% (depending on age and iron status), compared with 10–20% from infant formulas [19]. Most likely, the low content of iron in breast milk limits the role of iron as a crucial growth factor for microbial proliferation.

**Figure 1:**
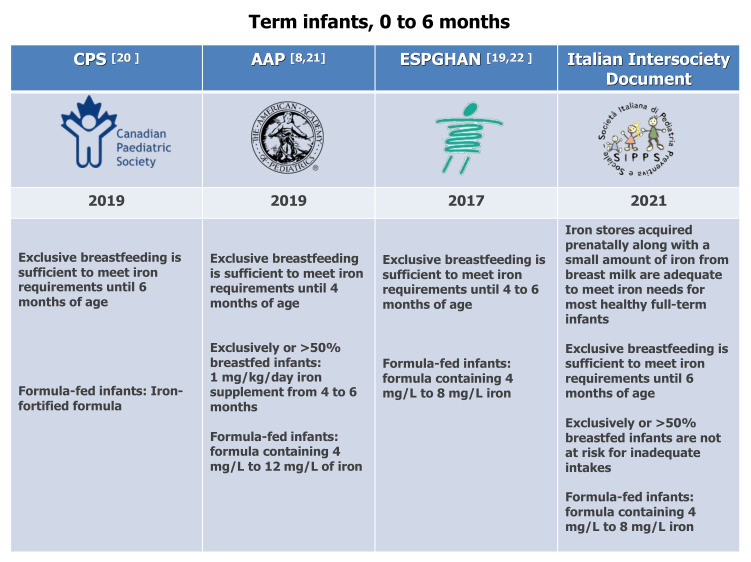
Dietary recommendations for term infants, 0 to 6 months. CPS [20], AAP [8,21], ESPGHAN [19,22].

Exclusively breastfeeding is sufficient to meet iron requirements up to 6 months of age, according to the Italian Document and the Canadian Pediatric Society (CPS) [8,20] or up to 4 to 6 months of age, according to the Committees on Nutrition of the AAP [8,21] and the European Society for Pediatric Gastroenterology, Hepatology, and Nutrition (ESPGHAN) [22] (Figure 1).

The AAP Committee on Nutrition recommends iron supplementation of 1 mg/kg/day at 4 to 6 months for exclusively or >50% breastfed infants. According to our expert panel, iron fortification should not be given to all infants. Instead, it should be given only to infants with definite ID or IDA because there are concerns about administering iron supplements to iron-replete infants, including delayed psychomotor development, impaired cognitive performance, poorer growth, and increased morbidity [23].

During infancy, impaired iron status could have deleterious effects on the gut microbiota, nervous system, and immune system that will persist into adulthood. Multiple pathologies, such as brain and tissue degeneration and inflammatory disorders, are associated with abnormal iron management. Whether iron deficiency in early childhood affects the regulation of other organ systems across the life span is being investigated. In the first months of life, disruption of the sophisticated mechanisms involved in iron absorption, storage and metabolism can lead to growth alterations and iron overload in the developing central nervous system (CNS) [24,25,26,27,28] It’s noteworthy that nowadays, there is still a lack of randomized controlled trials regarding the optimal iron supplementation in infancy and the long-term effects of infant formulas with different iron fortification on neurocognitive performance.

The molecular nature of iron metabolism and its transport to the brain is one of the still not well-understood aspects of iron cell biology. Iron accumulation can cause oxidative damage to complex brain structures, such as the hippocampus, with possible neurodegenerative effects [14,29]. The hippocampus, deeply embedded in the temporal lobe, plays an essential role in learning, consolidating short-term memories into long-term memories, and spatial navigation. The dysregulation of iron metabolism could impair these functions during the early stages of life [30].

Neonatal and infant gut microbial patterns are influenced by a variety of factors, such as the neonatal gestational age, the type of birth (vaginal vs. cesarean) and the location (home-born vs. hospital-born) of delivery, maternal microbiota, the number of siblings, the type of infant feeding (breast- vs. formula feeding), the timing and composition of complementary feeding, and drugs (proton pump inhibitors, antacids, antibiotics, and iron fortification) that may dysregulate infant physiology and lead to disease [31].

There is growing awareness that iron fortification during early infancy negatively affects the gut microbiota composition, decreasing lactobacilli and bifidobacteria, increasing enteropathogens (including *Salmonella* sp., *Clostridium difficile*, *Clostridium perfringens*, and pathogenic *Escherichia coli*) and intestinal inflammation (measured by fecal calprotectin) [32,33]. The effects of iron supplementation or fortification on the gut microbiota and intestinal inflammation in children are evident in low-income populations where hygiene standards are low, and the microbiota likely hosts pathobiont enterobacteria. Dysbiosis is an alteration to the microbial community structure and/or function that can drive the detrimental dysregulation of microbe–host homeostasis [34]. In recent years, it has been discovered that intestinal dysbiosis has a profound impact on metabolic and immune homeostasis. Alterations in an infant’s gastrointestinal tract microbiota have been associated with an increased risk of short- and long-term immunologically and metabolically mediated diseases.

The Committee on Nutrition of the AAP recommends starting formulas containing 4 mg/L to 12 mg/L of iron for term infants [8], whereas ESPGHAN [19,22] and the Italian inter-society Document recommend, for non-breastfed infants, formulas containing 4 mg/L to 8 mg/L iron (Figure 1).

Iron deficiency without anemia is the most common micronutrient deficiency worldwide. It is common in both low- and high-income countries, affecting an estimated 40% of children under five years of age [35,36]. There are no available statistics regarding ID in infants younger than one year of age [35]; nevertheless, recent National Health and Nutrition Examination Survey (NHANES) data show that 13.5% of children aged 1–2 years are iron-deficient [37]. Iron deficiency and anemia can usually result from a combination of increased iron loss and a decrease in intestinal absorption of iron and release from iron stores due to inflammation [38]. However, during infancy, dietary factors play a crucial role: exclusive breastfeeding for more than 6 months without iron supplementation and low bioavailability of complementary iron foods [39], high unfortified cow’s milk intake [40,41], and dietary restrictions (e.g., vegetarianism). Other known risk factors for iron deficiency in infancy include low socioeconomic status [42], preterm birth or birth weight <2500 g (due to low iron stores at birth) [43,44], increased iron requirements (e.g., rapid growth), infants born to mothers with obesity [45] or anemia or poor nutritional status [46], early umbilical cord clamping [47], and chronic infections.

## 3. Iron Status during the Complementary Feeding Period

Complementary feeding starts during a crucial stage of early life characterized by rapid growth and development, progressive perinatal iron stores depletion, a brain growth spurt, cognitive development, and the introduction to new foods.

The relationship between the timing of the introduction of complementary foods and growth, anthropometric parameters, body composition, and the likelihood of being overweight/obese remains unclear [48]. More randomized controlled trials that account for reverse causality and confounding factors (e.g., feeding modalities, breastfeeding versus formula feeding, baseline iron status, and different iron bioavailability in foods) are needed.

According to the Scientific Report of the 2020 Dietary Guidelines Advisory Committee, CF should not be started before the age of four months, and beginning CF at 4 to 5 months of age does not offer long-term benefits or drawbacks compared with starting at 6 months of age [49].

The expert panel of our Document found no significant difference between infants (breast or formula-fed) starting CF at 4–6 months of age and starting at 6 months of age in terms of short-term outcomes (iron status, growth) and long-term outcomes (the odds of being overweight/obese, type 2 diabetes mellitus, hypertension). Nevertheless, introducing complementary feeding before 6 months of age is not beneficial in healthy, full-term infants who live in developed countries. The Document concludes that complementary feeding should not be introduced before 6 months of age in healthy breastfed or formula-fed infants with regular bodyweight and stature (Moderate quality of evidence. Poor evidence to support a recommendation). The early introduction of complementary foods may be considered for healthy breastfed infants if the mother can no longer breastfeed at 4–6 months of age; however, infant formula is preferable, due to its more nutritionally balanced composition (Expert Opinion. Optional recommendation). Early complementary feeding may also be suggested for infants at high risk of iron deficiency [50].

The high prevalence of iron deficiency in infancy has led to the routine iron fortification of infant formulas and baby foods (regulated by the European directives 2006/125/CE and 2006/141/CE) that helps to reduce ID and IDA. However, the optimal amount of iron in these products, especially infant formulas, is still debated [24,51,52].

The human gut microbiota is considered to be a “bacterial organ system” that plays a central role in protecting against pathogens (direct killing, competition, enhancement of immune responses), regulating immune, metabolic, and endocrine functions, producing metabolites by the fermentation of dietary fibers and regulating bidirectional communication between the gastrointestinal tract and the central nervous system [53,54]. Although the composition of “healthy” gut microbiota is still a matter of debate, early “physiological” colonization is most likely to occur when the infant is born full-term by vaginal delivery and exclusively breastfed for 6 months. The “eubiotic” gut microbiota is also characterized by specific microbiological patterns, likely associated with health [55].

Various microorganisms have evolved complex transport and uptake systems to compete with the host for iron. Iron is essential for the growth and colonization of most pathogenic intestinal Gram-negative bacteria (enteropathogenic *Escherichia coli*, *Salmonella* sp., *Shigella* sp.). In contrast, iron-independent bacterial communities, such as Lactobacillus use manganese for their metabolism [56]. Therefore, iron overload (due to pharmacological supplementation and/or fortified complementary foods) could lead to gut dysbiosis in iron-replete infants.

Iron fortification trials in developing countries have led to safety concerns [57,58]. An international research team conducted two randomized, double-blind trials in 6-month-old Kenyan infants, providing iron-containing micronutrient powder (MNP) to weaning infants [33]. In the first trial, infants were given an MNP with 2.5 mg iron (as sodium iron etylenediaminetetracetic acid EDTA) or an MNP without iron; in contrast, in the second trial, infants were given a different MNP with 12.5 mg iron (as iron fumarate) or an MNP without iron. Comparing the microbiota composition before and after the supplementation, the authors observed a significant decrease in beneficial Bifidobacteria, an increase in pathogenic or potentially pathogenic microorganisms (*Salmonella *spp., *Clostridium difficile*, *Clostridium perfrigens,* and enteropathogenic *Escherichia coli*), and a higher frequency of episodes of diarrhea. Moreover, iron fortification increased fecal calprotectin levels, indicating intestinal inflammation. The research group concluded that iron fortification should not be given to all infants until safer formulas are available. Still, fortification should be administered only to infants with clear evidence of iron deficiency anemia while providing adequate protection against gastrointestinal side effects.

In a recent review, Michaelsen et al. [59] consider that more research is needed concerning the relationship between complementary feeding and gut microbiota. Current research shows the dominant role of nutrition compared to other possible factors in influencing the gut microbiota composition. Since deficient or excessive iron intakes can lead to impairment of the microbial ecosystems in early life, it has been suggested to establish an optimal range for iron fortification and supplementation aimed not only at providing adequate iron levels but also to maintain eubiosis, considering the importance of well-balanced microbial communities for the immune system and metabolic homeostasis later in life [60,61].

The collective microbial genome encodes for different metabolic substrates, with a central role in the immune and metabolic homeostasis of the host, thus improving its biochemical flexibility in response to substrate availability [62]. Recent interest in the potential gut-driven pathophysiologic pathways involved in gut dysbiosis led to attempts to strengthen this microbial ecosystem using the so-called “gut microbiota biomodulators” (Miniello) [34] (i.e., probiotics, prebiotics, synbiotics, and postbiotics).

Various studies found a correlation between gut microbiota biomodulators and an increase in iron availability because gut biomodulators can convert ferric iron (Fe^3+^) into ferrous iron (Fe^2+^), which the enterocytes can absorb more efficiently [63,64,65,66,67,68,69,70].

According to the definition of the International Scientific Association for Probiotics and Prebiotics (ISAPP), probiotics are “live microorganisms that when administered in adequate amounts confer a health benefit on the host” [71]. A recent systematic review and meta-analysis, examining the impact of probiotics on iron status and absorption, suggest that specific probiotic strains may be clinically valuable for optimizing dietary iron bioavailability, thereby improving iron status but not needing additional supplemental iron [72].

Human milk is the gold-standard food for newborns and growing infants to maintain their nutritional and healthy status. It contains the right balance of nutrients and confers many benefits due to its large group of bioactive compounds, including proteins/peptides, non-digestible oligosaccharides (human milk oligosaccharides (HMOs)), hormones, cells, mRNA, vitamins, nucleotides, minerals, and innate immune factors [73]. When breastfeeding is not possible, a suitable alternative is of great importance to satisfy infants’ nutritional needs. Infant formulas represent the sole alternative, and all industry efforts aim to resemble human milk composition. To mimic the benefits of breast milk, some formulas have even been supplemented with vegetable or animal non-digestible oligosaccharides with prebiotic activity (fructooligosaccharides (FOS), galacto-oligosaccharides (GOS), or a mixture of them), which are structurally different from those contained in human milk [74]. In particular, the prebiotic supplement GOS [71] is selectively used by commensal *Bifidobacterium spp*., and it has been shown to increase dietary iron absorption [68]. Recently some formulas have been enriched with two major HMOs, namely, 2′-fucosyllactose (2′FL) and Lacto-N-neotetraose (LNnT) [75,76].

Cytokines are signaling molecules that are involved in orchestrating innate and adaptive immunity, development, and differentiation of the immune system (including T helper cells polarization). It is essential to underline their role since the cytokine pattern of breastfed infants is notably different from that in formula-fed infants [77].

Goehring et al. investigated the presence of inflammatory biomarkers [75] in a subgroup of infants fed with formulas fortified with 2′-fucosyllactose (2′-FL), previously enrolled in a trial by Marriage et al. [78]. This research demonstrated that infants fed with formulas containing 0.2 g/L of 2′-FL and GOS presented significantly lower (29–83%) pro-inflammatory cytokines (IL-1α, IL-1 ra, IL-1β, IL-6, TNF-α) compared to infants fed with formulas containing only GOS. Instead, there were no significant differences between the cytokine patterns of infants fed with 2′-FL- and GOS-containing formula and breastfed infants. Commercial infant formulas do not contain the metabolic and immunity-boosting elements of breast milk. Still, the supplementation of infant formula with human milk 2′-FL could represent a crucial step toward achieving nutritional benefits similar to those guaranteed by human milk [79].

Upon validation of their mechanism of action, the gut microbiota biomodulators provide a proactive approach in optimizing iron metabolism during infancy and could beneficially modulate iron levels, avoiding pharmacological supplements.

Early-life nutrition is one of the most significant influencing environmental factors and can consistently impact development by regulating gene expression, even when the DNA is not altered. Preventative post-natal interventions from birth to 24 months are of pivotal importance and need to focus on dietary intake. In particular, the effects of high protein intake during childhood are still under debate: cohort trials have found an association between high protein intake (>15% of total kcal) in the first years of life and an increased risk of obesity and other chronic degenerative disorders in later life. According to recent epidemiological studies, a rapid increase in weight during the first two years of life and the following early adiposity rebound could have a long-term impact on the development of being overweight and of obesity [80]. Therefore, dietary prevention strategies should concern the intake of both macro- and micronutrients (i.e., proteins and iron). Most importantly, they should be personalized considering the type of milk, the CF composition, and practices. However, the effect of dietary proteins on growth trajectories during CF is still not clear [81].

Cow’s milk-based formula attempts to reproduce the nutritional composition of breast milk faithfully. Compared to breast milk, infant formula has a higher protein content [82]; however, cow’s milk (CM) is vastly different from breast milk and infant formulas: whole CM is characterized by a much higher protein concentration (34 g/L vs. 7–14/L in human milk) [83] and the composition of its proteins is also very different (~50–80%) compared to breast milk.

The timing of the introduction of unmodified CM is a crucial aspect to consider during infant feeding [84]. There is concern about whole CM consumption during the first year of life, due to possible adverse health consequences [83]. In particular, iron deficiency should be one of the main concerns when considering the benefits and disadvantages of unmodified cow’s milk vs. formula, such as low iron content, protein excess, occult intestinal blood loss, high renal solute load, and the presence of peptide β-casomorphin-7 [79]; the latter is particularly dangerous since its consumption can not only trigger a cascade of events that increase inflammation [85] but it could also harm neurogenesis and be involved in the development of different non-transmissible diseases [86]. Several mechanisms may contribute to iron deficiency in infancy, in terms of cow’s milk consumption; it can affect the iron status negatively by decreased non-heme iron absorption due to its high concentration of calcium and casein, or by occult intestinal blood loss, or by other unknown mechanisms, or by a combination them of all. Thus, unmodified unfortified cow’s milk should not be used during the first 12 months of life to avoid an excessively high protein intake and reduce the risk of consecutive overweight issues/obesity and iron deficiency. Limiting unmodified cow’s milk consumption has been suggested to maintain the overall diet protein content below 15% of the total calories [87].

**Figure 2:**
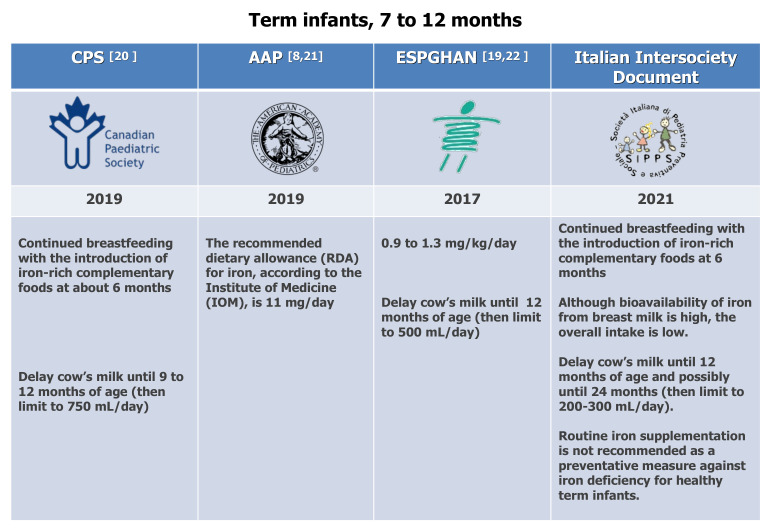
Dietary recommendations for term infants at 7 to 12 months. CPS [20], AAP [8,21], ESPGHAN [19,22].

Various studies have addressed the effects of drinking unmodified CM on children’s health, when breastfeeding and/or infant formula are not offered during the first year of life. Griebler et al. conducted a systematic review and meta-analysis focused on the health effects of CM consumption in term-born healthy infants of up to 3 years of age [88], taking into consideration patient-relevant outcomes (IDA) and surrogate parameters (iron status) when patient-relevant outcomes were not available. Overall, seven out of eight studies analyzed in this review showed a significantly higher risk of developing IDA in infants fed CM than those who received iron-fortified follow-on formula. The risk of bias limits the evidence base since some studies were conducted in families with low or meager socio-economic backgrounds, limiting their applicability to the general population. Nevertheless, the authors concluded that cow’s milk consumption in infancy is associated with an increased risk of developing iron-deficiency anemia. It is thus recommended that unmodified, unfortified CM should not be fed to infants, and toddlers should consume it only in modest quantities. The Italian expert panel recommends delaying the introduction of cow’s milk until 12 months and possibly until 24 months (depending on infant diet composition), limiting its intake to 200–300 mL/day. Higher intakes may displace iron-rich foods and contribute to increasing the daily protein load (Expert Opinion. Optional recommendation) (Figure 2).

Iron metabolism and uptake mechanisms are the most complex and strictly regulated among all micronutrients. Iron uptake depends on dietary composition (heme and non-heme iron), iron requirements, and host-related factors. Dietary iron can be found in two forms: heme iron, obtained from hemoglobin and myoglobin found in animal source foods, and non-heme iron (inorganic Fe^2+^ and Fe^3+^), contained mainly in plant-based foods [89]. Heme iron is well absorbed (up to 25%) and highly bio-available; thus, it is the preferred source of dietary iron. However, most of the iron in children’s diets is non-heme iron. Indeed, the proportion of heme iron in the overall level of dietary iron is often low, at approximately 10% in children of meat-eating populations and even lower in low-income countries or among the children of vegetarian peoples.

Although plenty of the literature has demonstrated that high amounts of animal proteins (especially dairy proteins) during the second semester of life can harm the infant metabolism, currently, it is still not possible to draw a definitive conclusion as to whether different protein sources introduced during complementary feeding (i.e., dairy vs. meat vs. plant) could induce distinct growth patterns [90]. Moreover, according to Tang, eubiotic gut microbiota might mediate protein quality and growth trajectories [80].

During complementary feeding, iron is mainly supplied through formula or breast milk, fortified cereals, and meat, which guarantees an optimal absorption due to its highly bio-available heme iron content. Even though Abrams et al. recommended increasing the proportion of heme iron in animal products where feasible [91], it is essential to remember that the protein intake must be below 15% of the total energy intake up to 2 years old [81].

The National Academia of Medicine assessed the Dietary Reference Intakes (DRIs) for iron, including the estimated average requirement (EAR), set at 6.9 mg/day, and the recommended daily allowance (RDA) set at 11 mg/day [92].

Using the Feeding Infants and Toddlers Study 2016 database [93], Abrams et al. [91] recently calculated the amount of consumed and daily absorbed iron among infants aged from 6 to 12 months, fed with different types of milk (breast, formula, or mixed feeding), considering the different iron bioavailability among dietary iron sources. The physiologic absorbed iron requirement was estimated to be 0.69 mg/day by applying a 10% absorption rate to the iron EAR (6.9 mg/day). The percentage of infants with iron intakes below the EAR, and the percentage of infants with calculated absorbed iron intakes below the estimated daily absorbed iron requirement (0.69 mg/day), were also calculated. The results showed that the calculated daily iron absorption was significantly below the recommended amount in exclusively breastfed infants and mixed-fed infants; actually, human milk iron has high bioavailability, but its concentration is modest.

According to Greer, the iron status of breastfed infants between the ages of 4 and 12 months should be monitored through suitable biomarkers that can distinguish ID from IDA [94]. Therefore, it is helpful to complete the laboratory iron panel including parameters such as serum transferrin, reticulocyte hemoglobin content, and soluble transferrin receptors (sTfR), which are indicative of either compromised iron status or iron redistribution.

The Italian intersociety Document recommends that complementary foods first introduced at about 6 months of age should be iron-rich (e.g., meat, meat alternatives, and iron-fortified cereals) and that formula for term infants should contain iron in the range of 4–8 mg/L, following ESPGHAN recommendations. (Figure 1) These recommendations were conceived taking into consideration the Scientific Report of the 2020 Dietary Guidelines Advisory Committee, which stated that foods and drinks high in iron could help maintain adequate iron status and prevent iron deficiency during the first year of life among infants with insufficient iron stores or in breastfed infants who do not receive sufficient iron from other sources [48].

The ESPGHAN position paper on CF stated that: “Because the composition and health effects of breast milk differ from those of infant formula, on a theoretical basis, it may seem sensible to give different recommendations on CF to breastfed versus formula-fed infants. Despite these theoretical considerations, devising and implementing different recommendations for the introduction of solid foods for breastfed infants and formula-fed infants may, however, present practical problems and confuse caregivers” [2]. The SIPPS/FIMP/SIDOHaD/SINUPE document disagrees with the last sentence since, firstly, it is essential to give different recommendations regarding CF, taking into account the type of milk feeding. Secondly, this statement is not based on scientific evidence, and further studies have not confirmed these concerns.

Micronutrient imbalance regarding iron, vitamin D, alpha-linolenic acid, and docosahexaenoic acid is common in European infants and young children, compared to the dietary reference values [95,96,97]. Moreover, transition to the family diet should be considered at a specific time when dietary care needs to control the amount of protein since the current protein intake in children of 12–36 months old is high [98]. There is compelling evidence demonstrating that higher protein intakes during the second year of life are likely to increase the propensity to become fat in childhood [99].

Breastfeeding rates fall with increasing age. Only a small proportion of infants are breastfed after 12 months of age; cow’s milk continues to be a significant determinant of total energy and protein intake. As recommended by the WHO, breastfeeding should ideally continue beyond infancy. If the infant is no longer breastfed after 12 months of age, unfortified cow’s milk or young child formula (YCF, also called “growing-up milk”) are the first-line milk alternatives. Different opinions have been expressed on whether YCF has any nutritional benefits over CM within the complete family diet, during the nutritionally vulnerable period of 1–2 years of age [100,101].

A double-blind, randomized placebo-controlled trial [102] was conducted on healthy toddlers aged 12 to 36 months to investigate the benefits of YCF (containing 12 mg/L iron and 17 μg/L vitamin D) compared to unmodified CM (containing 0.02 mg/L iron and no vitamin D). After 20 weeks, the probability of developing iron deficiency and vitamin D deficiency was significantly inferior in toddlers consuming YCF.

Another double-blind, randomized control trial was recently conducted on one-year-old children who, for 12 months, randomly received a YCF with reduced protein content (1.7 g protein/100 mL) or a non-fortified CM (3.1 g protein/100 mL) [103]. The researchers found, in the enrolled well-nourished children of two years of age, that the total protein intake (particularly protein intake from CM) increases insulin-like growth factor 1 (IGF-1) concentrations relative to the children’s total energy intake.

Based on the available evidence, ESPGHAN does not recommend routinizing the consumption of YCF in children from 1 to 3 years of age [100]. Nevertheless, YCF can be used as part of a strategy to increase the intake of iron, vitamin D, and polyunsaturated fatty acid and decrease protein intake, compared with unfortified CM. Therefore, the consumption of YCF could optimize protein and micronutrient intakes to meet the nutritional recommendations.

## 4. Conclusions

In the evolutionary process, the human body has developed sophisticated mechanisms of absorption and metabolism for the utilization of iron but not for its elimination. The complementary feeding period is critical for the martial state of the infant since there are concerns about both iron deficiency and excess. Therefore, complementary feeding should start at 6 months of age in both bottle-fed and breast-fed infants and then be customized according to the composition of meals and the basal iron status during the first six months of life, aiming to realize a tailor-made diet and prevent iron deficiency and iron-deficiency anemia.

“Leave your drugs in the chemist’s pot if you can heal the patient with food.”

Hippocrates

## Data Availability

Not applicable.
